# Comparative microbiomes of ticks collected from a black rhino and its surrounding environment

**DOI:** 10.1016/j.ijppaw.2019.05.008

**Published:** 2019-05-28

**Authors:** Seogwon Lee, Ju Yeong Kim, Myung-hee Yi, In-Yong Lee, Robert Fyumagwa, Tai-Soon Yong

**Affiliations:** a Department of Environmental Medical Biology, Institute of Tropical Medicine, Arthropods of Medical Importance Resource Bank, Yonsei University College of Medicine, Seoul, 03722, South Korea; b Brain Korea 21 PLUS Project for Medical Science, Yonsei University College of Medicine, Seoul, 03722, South Korea; c Tanzania Wildlife Research Institute, P.O. Box 661, Arusha, Tanzania

**Keywords:** Black rhino, *Diceros bicornis*, Ticks, *Amblyomma gemma*, Microbiome, Metagenomics

## Abstract

‘Eliska,’ an endangered black rhino (*Diceros bicornis*), died suddenly in Mkomazi National Park in Tanzania in 2016. Three *Amblyomma gemma* ticks were collected from Eliska's body, and four ticks were collected from the surrounding field. We conducted 16S rRNA targeted high-throughput sequencing to evaluate the overall composition of bacteria in the ticks' microbiomes and investigate whether the ticks could be the cause of Eliska's death. The ticks collected from Eliska's body and the field were found to differ in their bacterial composition. *Bacillus chungangensis* and *B. pumilus* were the most commonly found bacteria in the ticks collected from the field, and *B. cereus* and *Lysinibacillus sphaericus* were the most commonly found in the ticks collected from Eliska's body. The abundance was higher in the ticks collected from the field. In contrast, the equity was higher in the ticks collected from Eliska's body. No known pathogenic bacteria that could explain Eliska's sudden death were found in any of the ticks. The differences between the microbiome of ticks collected from Eliska's body and from the field indicate that the microbiome of ticks' changes through the consumption of blood.

## Highlights

• Ticks were collected from a dead Black rhino of the endangered species *Diceros bicorn.*
• Ticks collected from the body and the surrounding field had different microbiome patterns.
• *Bacillus chungangensis* and *B. pumilus* were commonly found in the ticks collected from the field, and *B. cereus* and *Lysinibacillus sphaericus* were commonly found in the ticks collected from Black rhino.

## Graphical abstract

**Figure:**
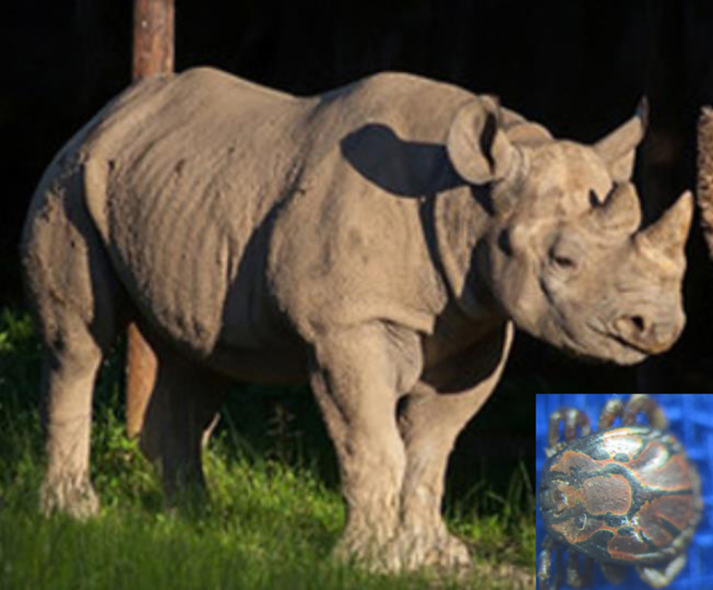

## 1 Introduction

Tick-borne diseases are caused by infectious agents transmitted through tick bites. Tick-borne diseases may be caused by rickettsia or other types of bacteria, as well as viruses and other pathogens (de la Fuente et al., 2017). A previous study reported that the geographic distribution and genotypes of *Coxella burnetii* and rickettsia differed among the different tick species that occur in Ethiopia, which suggests that there may be patients with tick-borne diseases of unknown etiology in this country (Kumsa et al., 2014, 2015). Tick-borne diseases cause major problems in livestock and wild animal health, especially in sub-Saharan Africa (Brites-Neto et al., 2015). There are many species of tick species including *Amblyomma gemma, Rhipicephalus appendiculatus* found on livestock and wild animals in Tanzania, and pathogens such as *Anaplasma marginale and Babesia bigemina* were found in the ticks (Fyumagwa et al., 2009; Kim et al., 2018b).

Black rhinos (*Diceros bicornis*) are critically endangered. In 2012, Eliska, a female black rhino, was born in Dvur Kralove Zoo in the Czech Republic, and moved to Mkomazi National Park in Tanzania. Although the average lifespan of black rhinos is between 35 and 50 years, Eliska died suddenly in 2016 (AWF, 2013). Ticks were found on Eliska's dead body, and these were collected and sent to the laboratory of the Arthropods of Medical Importance Bank, Yonsei University College of Medicine, Seoul, Korea, where we identified the species of ticks. We further analyzed the tick microbiomes using 16S rRNA targeted high-throughput sequencing to evaluate whether the ticks had any pathogenic bacteria that could explain Eliska's sudden death. We also collected and analyzed several ticks from the field where Eliska lived in order to compare the microbiome of ticks collected from the field and from Eliska's body.

## 2 Materials and methods

### 2.1 Tick collection from Eliska's body and the surrounding field

Simultaneously, three fully engorged ticks were collected from Eliska's dead body and four ticks were collected from the field surrounding the body (−4.094504, 38.122419 in World Geodetic System (WGS84)) in Mkomazi National Park, in Tanzania, in 2016. The four field samples were collected by flagging. All ticks used in this study were in their adult stage. Each ticks' surface was sterilized using alcohol immediately after collection, and the ticks were then individually stored. The samples were transferred to the laboratory in Korea, and their surfaces were again washed with alcohol. The ticks were then individually crushed and DNA immediately extracted.

### 2.2 Morphological identification of ticks

The ticks were identified as *Amblyomma gemma* based on morphology under a stereomicroscopic (Stemi DV4, Korea). Morphological characteristics of *A. gemma* include flat eyes and fine connections between central and lateral spots. *Amblyomma gemma* specimens also have medium size punctuations in the anterior area of the scutum (Walker et al., 2013). All ticks were in their adult stage, and the ticks collected from Eliska's body were fully engorged (Fig. S1).

### 2.3 DNA extraction from ticks

Total DNA was obtained using the NucleoSpin DNA Insect Kit (Macherey-Nagel, Germany) following the manufacturer's instructions. Each tick sample was separately placed in a bead tube and submitted to the following steps: cell lysis, binding of the DNA to the silica membrane, washing and then drying of the silica membrane. The DNA extracted from each sample was eluted in 20 ㎕ of the elution buffer. The entire processing and sequencing of the samples was conducted at a clean bench, under a sterilized hood, and in a DNA-free room. Autoclaved 200 ㎕ and 1000 ㎕ tips (Chembio, Korea) were used. DNA concentration was quantified using Nanodrop (Thermo ND-1000, USA). The extracted DNA was stored at −80 °C in a deep freezer.

### 2.4 Amplification of 16S rRNA by polymerase chain reaction (PCR)

The V3–V4 region of 16S rRNA was amplified by PCR using the following primer pair: forward primer, 5′-TCGTCGGCAGCGTCAGATGTGTATAAGAGACAGCCTACGGGNGGCWGCAG-3′; reverse primer, 5′-GTCTCGTGGGCTCGGAGATGTGTATAAGAGACAGGACTACHVGGGTATCTAATCC-3′ (Illumina MiSeq V3 cartridge [600 cycles]; Illumina, USA) (Kim et al., 2018a).

### 2.5 Next-generation sequencing (NGS)

A limited-cycle amplification step was performed to add multiplexing indices and Illumina sequencing adapters. Libraries were normalized, pooled, and sequenced on the MiSeq platform (Illumina MiSeq V3 cartridge [600 cycles]; Illumina) in accordance with the manufacturer's instructions.

### 2.6 Bioinformatics and statistics

Bioinformatic analyses were performed following previously described methods (Yoon et al., 2017; Kim et al., 2018a). Raw reads were processed through a quality check, and low quality (<Q25) reads were filtered using Trimmomatic 0.32 (Bolger et al., 2014). Paired-end sequence data were subsequently merged using PandaSeq (Masella et al., 2012). Primers were then trimmed using the ChunLab in-house program (ChunLab, Inc., Seoul, Korea), applying a similarity cut-off of 0.8. Sequences were denoised using the Mothur pre-clustering program, which merges sequences and extracts unique sequences, allowing up to two differences between sequences (Schloss et al., 2009). The EzBioCloud database (https://www.ezbiocloud.net/) (Yoon et al., 2017) was used for the taxonomic assignment using BLAST 2.2.22, and pairwise alignments were generated to calculate similarity (Myers and Miller, 1988; Altschul et al., 1990). The UCHIME algorithm and non-chimeric 16S rRNA database from EzTaxon were used to detect chimeric sequences for reads with a best hit similarity rate of <97% (Edgar et al., 2011). In ChunLab, contigs and singletons, which were identified when similarity was under 97% in the taxon assignment stage, were detected as chimeras based on the non-chimera data base (DB) of the corresponding region. The DB herein used was based on various databases such as NCBI and the ChunLab DB. The bioinformatics ‘usearch’ tool in ChunLab was used to directly remove chimeric reads. Sequence data were then clustered using CD-Hit and UCLUST (Edgar, 2010; Fu et al., 2012).

All of the described analyses were performed with BIOiPLUG, a commercially available ChunLab bioinformatics cloud platform for microbiome research (https://www.bioiplug.com/). Rarefaction for the obtained operational taxonomic units (OTUs) was calculated using the ChunLab pipeline, following Heck et al. (1975). The reads were normalized to 16,000 to perform the analyses. We computed the Shannon index (Shannon et al., 1948), unweighted pair group method with arithmetic mean (UPGMA) clustering (Sneath and Sokal, 1973), principal coordinates analysis (PCoA) (Gower, 1966), and permutational multivariate analysis of variance (PERMANOVA) (Anderson, 2001) based on the generalized UniFrac distance (Lozupone and Knight, 2005). We used the Wilcoxon rank-sum test to test for differences in the number of OTUs and used the Shannon index to compare microbiome diversity between the two groups of ticks (collected from Eliska's body and the field). We used linear discriminant analysis effect size (LEfSe) analysis to identify significantly different taxa between the two groups of ticks (Segata et al., 2011).

## 3 Results

**Fig. 1:**
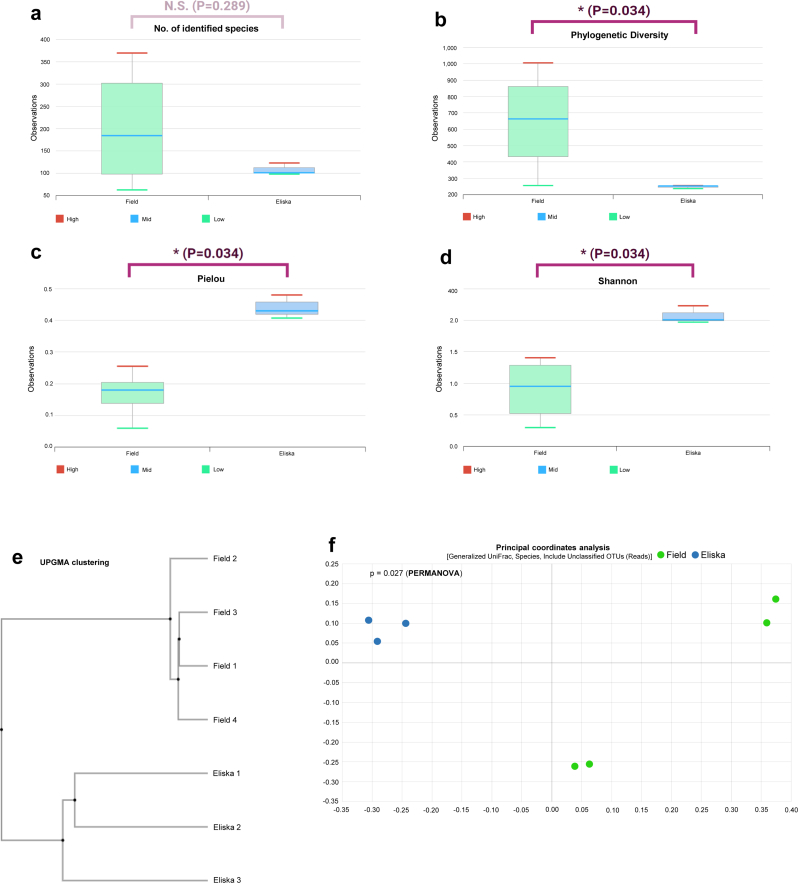
Box plots showing the alpha diversities (measurement of species richness and evenness within a habitat unit) of: **(a)** the number of operational taxonomic units (OTUs) found in microbiome taxonomic profiling (MTP); and **(b)** phylogenetic diversity (abundance); **(c)** Pielou diversity (equity); and **(d)** Shannon diversity (measurement of richness and equity in the distribution of species) among the samples from Eliska. Bars indicate the median, and the hinges represent the lower and upper quartiles. In panel **(a)**, there are no statistically significant differences between the two groups. However, in panels **(b)**, **(c)**, and **(d)**, there are statistically significant differences between the two groups. **(e)** Unweighted pair group method with arithmetic mean (UPGMA) clustering, and **(f)** principal-coordinate analysis depicting differences in taxonomic compositions of bacterial communities in *Amblyomma gemma* samples collected from Eliska's body and from the field. * indicates statistical differences between the two groups of ticks (Wilcoxon rank-sum test, p < 0.05).

Based on their morphological characteristics, all ticks were identified as *Amblyomma gemma*. The average reads assigned to bacteria were 20,091 reads assigned to 110 species (OTUs) for the ticks collected from Eliska's body. For the ticks collected from the field, the average reads assigned to bacteria were 64,687 reads assigned to 260 species (Table S1). The rarefaction curve of all samples formed a plateau (Fig. S2). The number of OTUs was not significantly different between the two groups of ticks (Fig. 1a). The phylogenetic (abundance) index was significantly higher for the ticks collected from the field than for the ticks collected from Eliska's body (Fig. 1b, p = 0.034). In contrast, the Pielou (equity) and Shannon (abundance and equity) indexes were significantly higher in the ticks collected from Eliska's body than in ticks collected from the field (Fig. 1c and d, p = 0.034).

The UPGMA clustering showed that the ticks were organized according to group; ticks collected from Eliska's body are mainly clustered at the bottom of the diagram in Fig. 1e. The PCoA results indicate that the ticks collected from Eliska's body were more closely distributed than those collected from the field (Fig. 1d). This indicates that the ticks collected from Eliska's body shared a greater similarity in bacterial composition than the ticks collected from the field (Fig. 1f). Moreover, a significant difference in microbiome composition between the two groups of ticks was detected using PERMANOVA. This analysis confirmed that the fact that ticks were collected from Eliska's body was a significant factor in determining microbiome composition (p = 0.027). PERMANOVA is a non-parametric statistical test for differences between multivariate datasets in the centroid or dispersion of groups (Ericsson et al., 2018).

**Fig. 2:**
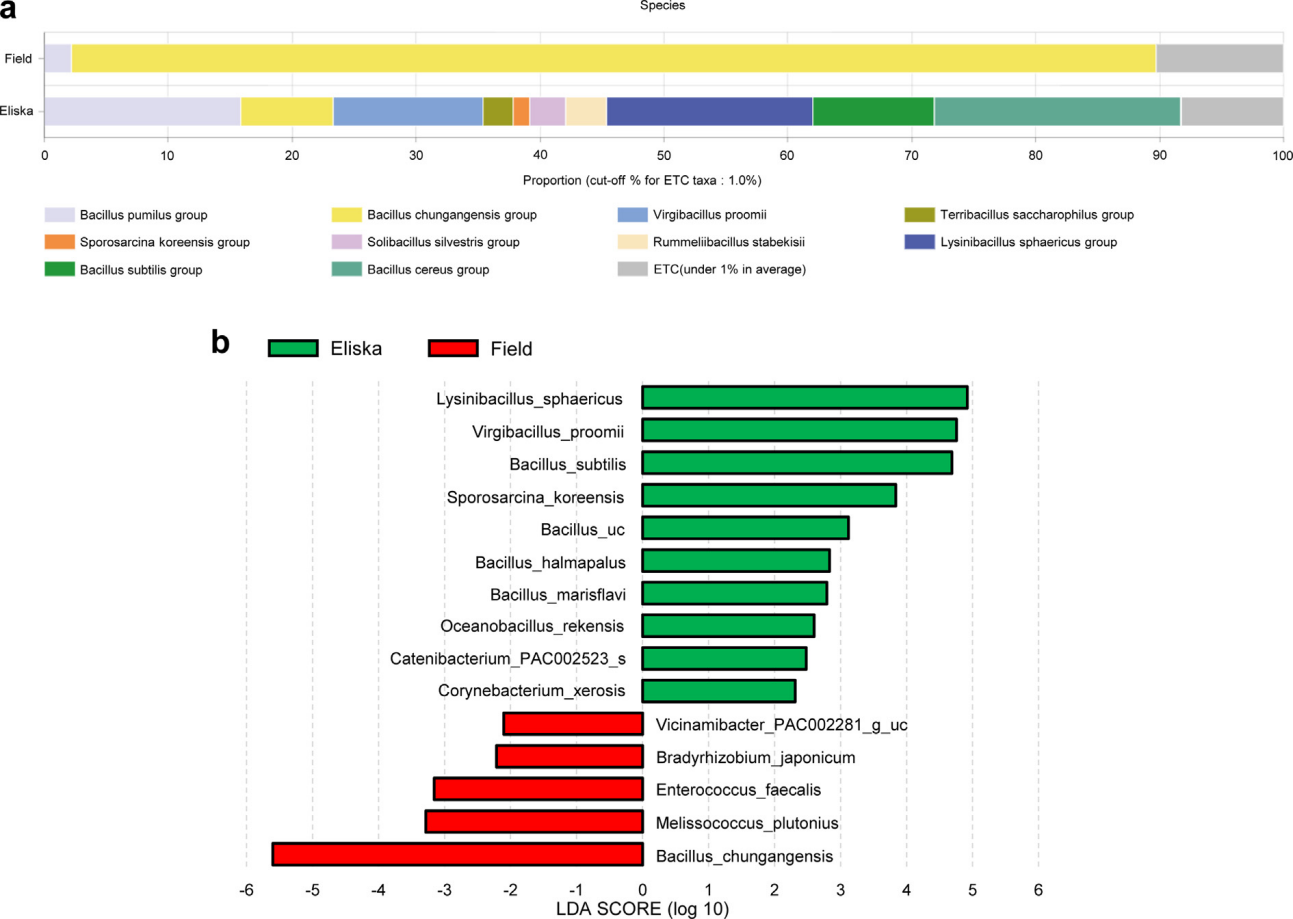
**(a)** The distribution of bacterial taxa at the species level in tick samples collected from Eliska's body and from the field. Each bar depicts the mean relative abundance value of independent replicates. Species comprising more than 1% of reads are shown. Each bar depicts the mean relative abundance value of independent replicates (n = 4 ticks collected from the field; n = 3 ticks collected from Eliska's body). **(b)** LEfSe analysis of differentially abundant bacterial taxa between ticks collected from Eliska's body and ticks collected from the field. Only taxa meeting an LDA significant threshold of >2 are shown.

Regarding the bacterial taxa found in the two groups of ticks (Table S2), at the species level, four species of *Bacillus* accounted for 55.95% and 89.67% of the total reads in ticks collected from Eliska's body and the field, respectively. In ticks collected from the field, bacteria that accounted for more than 1% of the total reads included *B. chungangensis* (87.14%) and *B. pumilus* (2.17%). In ticks collected from Eliska's body, bacteria that accounted for more than 1% of the total reads included *B. cereus* (19.86*%), Lysinibacillus sphaericus* (16.64%), *B. pumilus* (15.82%), *Virgibacillus proomii* (12.10%), *B. subtilis* (9.81%), *B. chungangensis* (7.45%), *R. stabekisii* (3.28%), *S. silvestris* (2.87%), *Terribacillus saccharophilus* (2.41%), and *Sporosarcina koreensis* (1.38%) (Fig. 2a).

To identify significant differences in bacterial abundance between the two groups of ticks, a LEfSe analysis was performed. The highest LDA scores found in the ticks collected from Eliska's body were for *L. sphaericus* (4.92) and *V. proomii* (4.76). In the ticks collected from the field, *Melissococcus plutonius* (−3.28) and *Enterococcus faecalis* (−3.16) were the species with the highest LDA score (Fig. 2b).

## 4 Discussion

*Amblyomma gemma* is abundant in dry areas and is mainly distributed in the northeast of Tanzania (Lynen et al., 2007). Hosts of *A. gemma* include herbivores, such as giraffes and buffalo, but adult *A. gemma* also use cattle and camels as hosts. *Amblyomma* species are responsible for transmissions of viruses and bacteria to animals, including humans (Sang et al., 2006).

We evaluated the microbiomes of *A. gemma* collected from Eliska's body and from the surrounding field, to check whether the bacterial profiles differed between the two groups of ticks. Regarding alpha diversity analyses, the phylogenetic (abundance) index was higher in ticks collected from the field than in those collected from Eliska's body; however, the Pielou (equity) and Shannon (abundance and equity) indexes were significantly higher in the ticks collected from Eliska's body. This result indicates that the number of bacterial species (OTUs) in the field group was high but that equity was low, as few species (*B. pumilus* and *B. chungangensis*) accounted for approximately 90% of the microbiome of the group. Similarly, there were differences in bacterial composition between ticks collected from Eliska's body and ticks collected from the field in clusters in PCoA (generalized UniFrac).

*Rickettsia, Anaplasma, Ehrlichia, and Bartonella* are pathogenic bacteria that can exist in ticks (Khoo et al., 2016). However, none of these bacteria were found in the ticks included in this study. *Coxiella*, *Rickettsiella*, and *Wolbachia*, which are known endosymbionts, were also not found. However, *Bacillus* spp., the environmental bacteria, which have been reported in other studies, were abundant in the ticks in this study. Other environmental bacteria such as *Sphingomonas*, *Pseudomonas,* and *Staphylococcus* have also been reported in ticks (Khoo et al., 2016; Trout Fryxell and DeBruyn, 2016). Therefore, we conclude that Eliska's death was not caused by any known pathogenic bacterial infection transmitted by ticks.

In the LefSe analysis, we identified the characteristics of the bacteria that were significantly different between the two groups. In the ticks collected from Eliska's body, *L. sphaericus* and *V. proomii* were more prevalent. *Lysinibacillus sphaericus* is a bacterium that is toxic to mosquitoes. However, there have been no reports yet of *L. sphaericus* causing harm to humans or other mammals (Berry, 2012). *Virgibacillus proomii* enhances leukocyte phagocytic activity in fish, and increases fish survival by activating immune defenses (Salinas et al., 2005). On the other hand, in the ticks collected from the field, *M. plutonius* and *E. faecalis* were more prevalent. *Melissococcus plutonius* is morphologically similar to *Enterococcus* (Ansari et al., 2017). *Melissococcus plutonius* causes European foulbrood, a biological disease that affects and kills the larvae of honeybees (Budge et al., 2014). *Enterococcus faecalis* is a gram-positive bacterium that lives in the gastrointestinal tract of humans or other mammals (Ryan and Ray, 2004). *Enterococcus faecalis* is found in most healthy individuals, but can cause various diseases in humans, such as endocarditis and meningitis (Pintado et al., 2003).

A previous study reported that the diversity of bacteria of *Amblyomma americanum* increased when the ticks consumed blood meal (Heise et al., 2010). Our experimental data also indicate that the Shannon diversity was higher in ticks collected from Eliska's body, which had been feeding on the rhino's blood.

When *Ixodes pacificus* ticks feed on the blood of lizards, their microbiome a change and increase their resistance to *Borrelia burgdorferi*, a pathogen. This indicates that, in natural systems, blood meal can affect the microbiomes of ticks, which in turn can affect pathogen transmission (Swei and Kwan, 2017). In this study, the differences between the microbiome of ticks collected from Eliska's body and from the field indicate that the microbiome of ticks' changes through the consumption of blood. Blood molecules may regulate growth of specific bacteria. For example, D-alanine from blood can regulate the gram-positive bacterial biofilm formation by interrupting *Ixodes scapularis* antifreeze glycoprotein (IAFGP) in *Ixodes scapularis* (Abraham et al., 2017). Therefore, in the present study, changes in the *A. gemma* microbiome resulting from the activity of blood molecules were expected. As Eliska's blood was not collected, we were unable to search for bacteria in it, which is a limitation of this study. Nevertheless, given that no pathogenic agent was found in ticks from Eliska and that mammalian blood usually has no bacteria, we believe that the microbiomes identified in the ticks were not from Eliska's blood.

## 5 Conclusions

In conclusion, the microbiomes of ticks collected from a black rhino and its surrounding environment were investigated using 16S rRNA targeted high-throughput sequencing. There were significant differences between the two groups of ticks, which possibly resulted from the consumption of blood in the group of ticks collected from the rhino's body. No pathogenic bacteria were found in any of the ticks that could explain the sudden death of this black rhino.

## Conflicts of interest

The authors have no conflict of interest.

## Figures and Tables

**Amblyomma gemma female.JPG:**
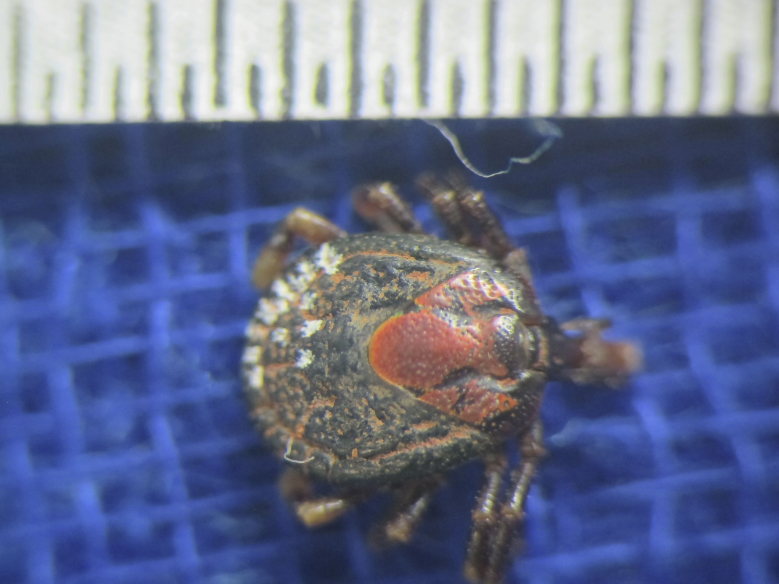


**Amblyomma gemma male.JPG:**
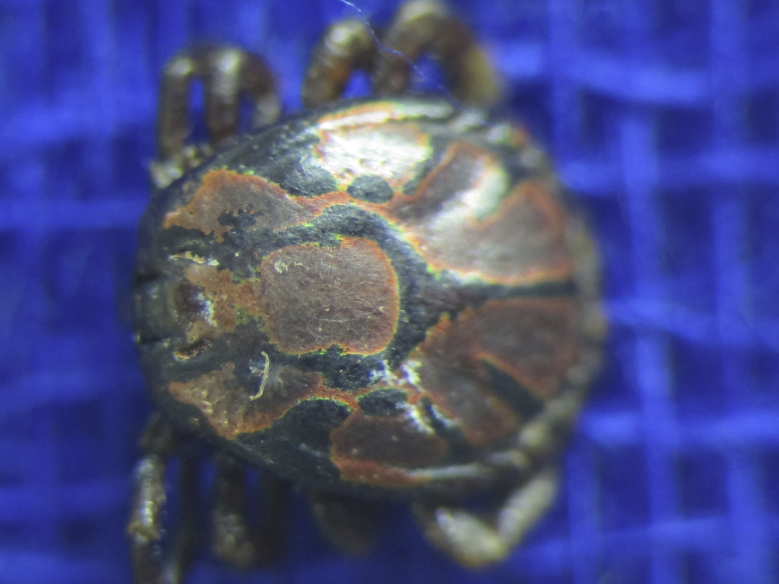


**Rarefaction curves _spl_Eliska_spl_.JPG:**
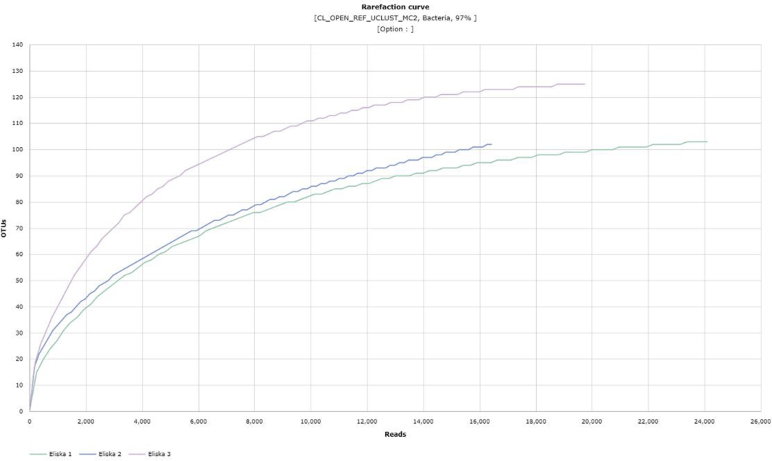


**Rarefaction curves _spl_Field_spl_.JPG:**
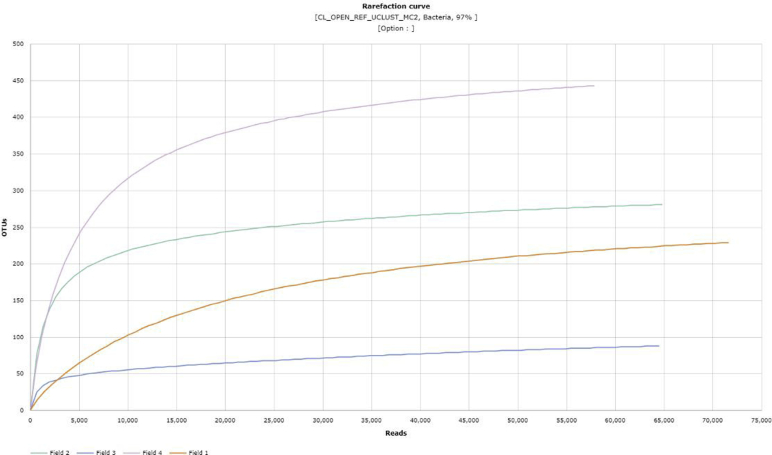


## Appendix A Supplementary data

Supplementary data legends.docx Supplementary data legends.docx

Table S1. Frequency charts and Shapiro-Wilk test.xlsx Table S1. Frequency charts and Shapiro-Wilk test.xlsx

Table S2. List of taxa.xlsx Table S2. List of taxa.xlsx
