# Supplementary material for: Comparative microbiomes of ticks collected from a black rhino and its surrounding environment
Source: Int J Parasitol Parasites Wildl. 2019 May 28;9:239–43. doi: 10.1016/j.ijppaw.2019.05.008 (PMC6556756; doi:10.1016/j.ijppaw.2019.05.008)
Supplement: Supplementary data legends.docx [file mmc1.docx]

**Figure S1.** Morphological identification of tick species under a stereomicroscopic. *Amblyomma gemma*: **(a)** female, **(b)** male. The ticks were in their adult stage.

**Figure S2.** Rarefaction curves of the number of operational taxonomic units (OTUs) of the two groups of ticks. **(a)** Rarefaction curves of the Eliska group. **(b)** Rarefaction curves of the field group.

**Table S1**. Frequency charts and Shapiro-Wilk test for the number of reads and genera detected in the two groups of ticks.

**Table S2.** List of taxa found in the two groups of ticks.
